# Supplementary material for: Learning curve and surgical outcome of robotic assisted colorectal surgery with ERAS program
Source: Sci Rep. 2022 Nov 29;12:20566. doi: 10.1038/s41598-022-24665-w (PMC9709162; doi:10.1038/s41598-022-24665-w)
Supplement: Supplementary file 2 — Supplementary Information 2. [file 41598_2022_24665_MOESM2_ESM.zip › RAL Raw data-Ñ[▒K/096/OP N_ñw╝╨░O▒Kñσ.pdf]

W95 / W95

臺中榮民總醫院
手術室護理記錄

[redacted]

基本資料

- 室別： 開刀房-17室，手術科別： CRS，手術類別： 常規，日期： 2021/07/08
- 進入時間： 1345 ； 麻醉開始時間： 1402 ； Time Out時間： 1450 ； 劃刀時間： 1451 ； 手術結束時間： 2050 送出時間： 2120 ；
- 麻醉方式： GE

  診斷： Malignant tumor of rectum + T COLON CA

  手術名稱： Robot Assisted Low Anterior Resection

  手術局部用藥： nil

  術前預防性抗生素：
    - 抗生素名稱一： Veterin 1GM IVD/由麻醉護理人員給予
    - 時間： 1440
    - 上次簽章: 歐真勤

  ，追加預防性抗生素：
    - 抗生素名稱一： Veterin 1GM IVD/由麻醉護理人員給予
    - 時間： 1840
    - 上次簽章: 林家羽
- 手術醫師： 蔣鋒帆
- 麻醉醫師： VS 齊光彪
-
    - 刷手護士A ： 林家羽，時間： 1345，刷手護士B ： 陳妍希，時間： 1345，接替刷手一： 陳家瑋，時間： 1500
    - 流動護士A ： 歐真勤，時間： 1345，接替流動一： 林家羽，時間： 1500

皮膚狀況

A-V Shunt： 無

術前： 完整

記錄者： 上次簽章: 歐真勤

術後： 不完整

- 1.紅腫

部位(術後)：

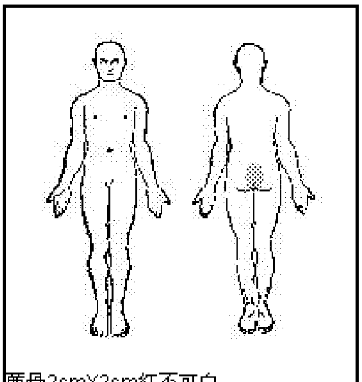

記錄者： 林家羽

手術準備

手術臥式 單一手術臥式

-
    - 手術臥式： 截石臥式
    - 使用附件： 溫毯 38 ℃，頭圈，墊肩，腳架
    - 皮膚準備： 10%Aq Betadine，Hibiscrub，2% Chlorhexidine
    - 電燒： 使用電燒: 電極片位置： 其它： 迴路導墊，雙極電燒，記錄者： 上次簽章: 歐真勤

止血帶T： 無

植入物

- 植入物名稱/號碼/數量/部位： Da Vinci Xi endowrist stapler 45 green*2/colon

, 記錄者： 上次簽章: 林家羽

標本送檢

- 細菌培養 無
- 細胞學 無
- 冰凍切片 無
- 病理標本
  組織名稱一： IMA root
  ,
  組織名稱二： distal cut end
  ,
  組織名稱三： A-colon +rectum
- 截肢 無

標本交付 標本： 3 袋 ， 標本： 0 盒 ， 標本： 0 瓶 ， 細胞學： 0 管 ， Culture Tube： 0 管 ， 被交付醫師： R 陳映晴 ，

- 記錄者： 上次簽章: 林家羽
- 時間： 2030

傷口術後敷料

傷口敷料： 傷口敷料： 無

- 請於圖上標示部位：

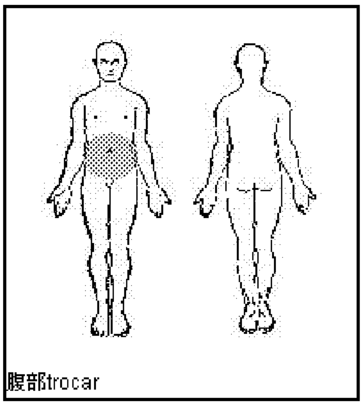

- 傷口敷料： 軟性敷料+紗布

導管

導管	天數	導管資訊	插入日期	插入時間	更換日期	拔除日期	拔除時間	拔除原因	預計拔除/更換	預計拔除/更換日期
A-line (1)	1	號碼:20 Ga; 部位:左手;	2021/07/08	1415						
Sump NG Tube(1)	1	號碼:16 Fr.; 深度:60cm; 部位:胃;	2021/07/08	1420						
Foley(1)	1	號碼:16 Fr.; 部位:膀胱; 註記:10cc;	2021/07/08	1410						
Penrose Drain(1)	1	號碼:12 mm.; 部位:骨盆腔;	2021/07/08	2000						
CVC(1)	1	號碼:8 Fr.; 深度:15cm; 部位:jugular (右); 象限:RU;	2021/07/08	1420						

用物計數

- 手術中出血量： minimal c.c.
- 手術中尿量： 550 c.c.
-
  - 其它： 無

用物計數： 多切口同時縫合

- 1.器械： 正確，2.縫針： 正確，3.刀片： 正確，4.紗布： 正確，5.腹部墊： 未使用，6.紗球： 未使用，7.PEANUTS ： 未使用，8.紗條： 未使用，9.絲帶： 未使用，10.針頭： 未使用，11.Vessel loop： 未使用，12.Boots： 未使用，13.Jelco： 未使用，14.棉條： 未使用，
  - 第一次：
    - 刷手護士 ： 陳家瑋
    - 流動護士： 上次簽章: 林家羽
    - 時間： 2015

  ,
  - 第二次：
    - 刷手護士 ： 陳家瑋
    - 流動護士： 上次簽章: 林家羽
    - 時間： 2025

  ,
  - 第三次：
    - 刷手護士 ： 陳家瑋
    - 流動護士： 上次簽章: 林家羽
    - 時間： 2035

備註

備註： davinci 30度 XI scopy:No.2

- 記錄者： 上次簽章: 歐真勤
- 時間： 1450

填寫護理人員 林家羽

正式摘要

W95 / W95

doctorRank:B
